# Temporal trends in patients with peripheral artery disease influenced by diabetes mellitus in Germany

**DOI:** 10.1111/1753-0407.13316

**Published:** 2022-09-22

**Authors:** Volker H. Schmitt, Lukas Hobohm, Markus Vosseler, Christoph Brochhausen, Thomas Münzel, Christine Espinola‐Klein, Karsten Keller

**Affiliations:** ^1^ Department of Cardiology University Medical Center Mainz (Johannes Gutenberg‐University Mainz) Mainz Germany; ^2^ German Center for Cardiovascular sdfsResearch (DZHK) Partner Site Rhine Main Mainz Germany; ^3^ Center for Thrombosis and Hemostasis (CTH) University Medical Center Mainz (Johannes Gutenberg‐University Mainz) Mainz Germany; ^4^ Institute of Pathology, University of Regensburg Regensburg Germany; ^5^ Medical Clinic VII, Department of Sports Medicine University Hospital Heidelberg Heidelberg Germany

**Keywords:** amputation, atherosclerosis, diabetes mellitus, epidemiology, health services research, peripheral artery disease, time‐trends, 糖尿病, 外周动脉疾病, 时间趋势, 截肢, 动脉粥样硬化, 流行病学, 卫生服务研究

## Abstract

**Introduction:**

In patients with peripheral artery disease (PAD) the presence of diabetes mellitus (DM) is associated with higher morbidity and mortality. Because huge efforts are made to improve medical care of patients with DM including chronic disease programs, the aim of the present study was to investigate temporal trends regarding the clinical burden of DM on PAD patients within a 15‐year observational period.

**Methods:**

We analyzed all patients hospitalized because of PAD between 2005 and 2019 in Germany stratified regarding DM.

**Results:**

Overall, 2 654 871 hospitalizations of PAD patients (865 823 with DM) were included. Hospitalizations based on PAD inclined from 142 778 in 2005 to 190 135 in 2019 (*β* 3956 per year; 95% confidence interval [CI] 3034–4878, *p* < .001) with simultaneous increase of hospitalizations of PAD patients with DM (2005: 41609 (29.1%) versus 2019: 65 302 (34.3%); *β* 2019 per year [95% CI 1593–2446], *p* < .001). Amputation rates (*β* −0.42 [95% CI −0.44 to −0.40]; *p* < .001) as well as in‐hospital case‐fatality rate (2005: 4.7%, 2019: 2.8%; *β* −0.64 [95% CI −0.69 to −0.59]; *p* < .001) decreased in diabetic PAD patients during the observational time. In spite of improved morbidity and mortality in the last years of the observational period, patients with DM still suffered from an increased risk for morbidity and mortality during the observational period compared to nondiabetic PAD patients.

**Conclusions:**

Despite the progress in DM treatments, DM still was associated with an unfavorable clinical patient profile and remained a substantial risk factor for morbidity and mortality in hospitalized patients with PAD and DM in Germany between 2005 and 2019.

## Graphical abstract

**Highlights**

- Annual numbers of hospitalizations of patients with peripheral artery disease (PAD) in coprevalence with diabetes mellitus (DM) increased significantly during the observational period from 2005 to 2019.
- Age and comorbidity‐burden of PAD patients with DM aggravated over time, whereas major adverse cardiac and cerebrovascular events and in‐hospital mortality of PAD patients with DM decreased from 2005 to 2019.
- The proportion of female PAD patients with DM decreased from 2005 to 2019.
- Annual numbers of amputation surgeries decreased from 2005 to 2019 driven by declining numbers of major amputations.
- Compared to nondiabetic persons, presence of DM was associated with poor in‐hospital outcome, which was widely unchanged over time.

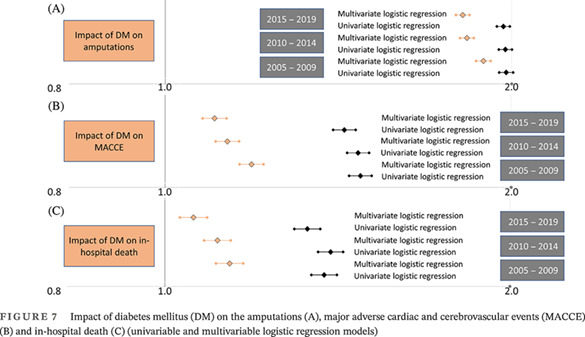

## 摘要

**背景:**

在患有外周动脉疾病(PAD)的患者中，糖尿病(DM)的存在与较高的发病率和死亡率相关。由于为改善DM患者的医疗保健(包括慢性病项目)付出了巨大努力，本研究旨在调查15年观察期内DM对PAD患者临床负担的时间趋势。

**方法:**

我们分析了2005年至2019年间德国根据DM分层的所有因PAD住院的患者。

**结果:**

共有2,654,871 例 PAD住院患者(865,823 例DM)纳入分析。基于PAD的住院人数从 2005 年的 142,778 人增加到 2019 年的 190,135 人(β 3956/年 [95%CI 3034‐4878]，P<0.001)，同时 PAD 合并 DM 患者的住院人数增加(2005 年:41,609 人(29.1%)vs. 2019 年:65,302 (34.3%), β 2019/年 [95%CI 1593‐2446]，P<0.001)。截肢率(β ‐0.42 [95%CI ‐0.44 to ‐0.40], P<0.001)以及住院病死率(2005:4.7%，2019:2.8%, β ‐0.64 [95%CI ‐0.69 to ‐0.59], P<0.001) 在观察期间糖尿病 PAD 患者降低。尽管在观察期的最后几年发病率和死亡率有所改善，但与非糖尿病PAD 患者相比，糖尿病患者在观察期内的发病率和死亡率风险仍然增加。

**结论:**

尽管DM治疗取得了进展，但DM仍然与不良的患者临床状况相关，并且仍然是 2005年至 2019 年间德国住院的 PAD 合并 DM 患者发病率和死亡率的重要危险因素。

## 1 INTRODUCTION

Diabetes mellitus (DM) represents an important risk factor for the development of organ damage and cardiovascular disease.^1^ As a concomitant disease, DM is associated with increased morbidity and mortality in various ailments.^2^, ^3^ The worldwide epidemic of DM with its increasing prevalence^4^ causes a high disease burden because of micro‐ and macrovascular alteration with resulting elevated risk for cerebrovascular and cardiovascular ischemic events as well as end organ damage, for example, of the kidney.^5^ Peripheral artery disease (PAD) is the clinical manifestation of atherosclerosis affecting the lower extremities leading to claudication and is accompanied by elevated risk for limb amputation.^6^ PAD is associated with increased loss regarding quality of life, morbidity, and mortality.^5^ Currently about 200 million people are affected by PAD worldwide^7^ and, similarly to DM, the prevalence of PAD is also increasing.^6^ In this context, DM represents a main risk factor for the development of PAD besides smoking and hypercholesterinaemia.^8^ Diabetic patients with coronary artery disease (CAD) were shown to have an increased risk also for consisting PAD with raising risk according to severity of CAD.^9^ Moreover, patients with PAD and concomitant DM were shown to have a less favorable clinical profile compared to PAD patients without DM and have consecutively a higher risk for death and morbidity including limb amputation.^10^

Regarding both, PAD and DM huge efforts have been made in the past decades to improve therapy approaches including the ongoing development of new medication pathways, the improvement of surgical and interventional procedures as well as the implementation of better patient education and medical surveillance strategies into the regular therapy regime to manage not only DM but also PAD. In disease management programs, patients are at constant control of the disease status with improvement of the therapy regime if necessary.^11^, ^12^, ^13^ Regarding the vast impact of DM on PAD and its negative influence concerning morbidity, amputation, and mortality outcome, patients with diabetes represent a group among PAD patients, which requires special attention regarding treatment care and prevention of disease progress.^10^ Owing to the immense efforts of the past years to improve outcome of PAD patients, time trend analyses are of crucial interest to evaluate achieved improvements and to identify ongoing challenges. In the present study, temporal trends of PAD patients with and without DM were analyzed in the German Nationwide Sample investigating all patients who were hospitalized between the years 2005 and 2019 because of PAD in Germany. Consecutively, our study represents a time trend assessment of diabetic and nondiabetic PAD patients within an observational period of 15 years.

## 2 MATERIALS AND METHODS

We analyzed all hospitalizations of patients with a main diagnosis of PAD (International Classification of Diseases [ICD] code I70.2) in Germany during the observational period between the years 2005 and 2019 (source: Research Data Center (RDC) of the Federal Statistical Office and the Statistical Offices of the federal states, diagnosis related groups (DRG) statistics 2005–2019, and own calculations). Patients' main diagnosis is defined as that diagnosis, which is mainly responsible for patients' hospitalization (admission to the hospital).^10^, ^14^

In Germany, patients' diagnoses are coded in accordance with the established coding guidelines ICD, 10th Revision with German Modification (ICD‐10‐GM); additionally, diagnostic, surgical as well as interventional procedures are coded by established surgery, diagnostic, and procedures codes (Operationen‐ und Prozedurenschlüssel [OPS codes]).^10^, ^15^, ^16^ The Federal Statistical Office of Germany (Statistisches Bundesamt, Wiesbaden, Germany) gathers all data from all inpatient cases in Germany coded and processed according to the DRG system.^10^

In the present study, we selected and included all hospitalizations of patients admitted because of PAD, who were identified by the ICD code I70.2 during the observational period 2005–2019 in Germany. The identified and included hospitalization cases with PAD diagnosis were stratified for the presence of DM (ICD codes E10–E14) and regarding the treatment year.

For the analyses, we subdivided the 15‐year observational period into three 5‐year cycles: the first period includes the years 2005–2009, the second period the years 2010–2014, and the third period comprises the years 2015–2019.

We analyzed the impact of DM on amputations, in‐hospital case‐fatality, and major adverse cardiac and cerebrovascular events (MACCE) in these PAD patients. Additionally, temporal trends in these patients regarding total numbers, outcomes, and patients' profile were investigated.

### 2.1 Study end points and in‐hospital adverse events

The primary study outcome was defined as in‐hospital death of all causes. The secondary study outcome comprised MACCE (composite outcome of all‐cause in‐hospital death, acute myocardial infarction [ICD code I21], and/or ischemic stroke [ICD code I63]). Furthermore, the frequency of amputations was assessed.

#### 2.1.1 Definitions

Obesity was defined in this study according to the recommendations of the World Health Organization as a body mass index ≥30 kg/m^2^.^17^ Shock as well as cardiopulmonary resuscitation (CPR) were defined in accordance with current European guidelines.^18^, ^19^, ^20^ In this study, major amputations were defined as surgeries with amputations above the ankle (OPS code: 5‐864) and minor amputations as surgeries comprising amputations below the ankle (OPS code: 5‐865). Amputations of the upper extremities and amputations for reasons other than limb ischemia, such as venous ulceration, trauma, and malignancy, were consistently not included in the present analysis.^10^, ^21^, ^22^

### 2.2 Ethical aspects

In accordance with German law, an approval by the ethical committee as well as an informed consent of the included patients were not required, because the present study did not involve a direct access of data of individual patients by the study investigators.

### 2.3 Statistical methods

Temporal trends of annual and age‐related hospitalizations of PAD patients and PAD patients with DM as well as relative mortality rate (case‐fatality rate), performed amputations and rate of adverse in hospital events, were calculated on an annual and age‐dependent (age‐decade) basis. Linear regressions were used to assess trends over time and the results are shown as beta (*β*) with corresponding 95% confidence intervals (CI).

As mentioned, we subdivided the 15‐year observational period into three 5‐year cycles (first cycle 2005–2009, second cycle 2010–2014, and third cycle 2015–2019) and compared the three periods. Descriptive statistical comparisons of PAD patients with DM of the three 5‐ear cycles were computed as absolute numbers and corresponding percentages and compared with the help of the Kruskal–Wallis test.

The third main part comprised the analysis regarding the impact of DM on in‐hospital adverse events and in‐hospital death in PAD patients in the three different 5‐year cycles performed by the use of univariable and multivariable logistic regression models. The results were presented as odds ratio (OR) and 95% CI. The multivariable regression models were adjusted for age, sex, obesity, cancer, heart failure, coronary artery disease, chronic obstructive pulmonary disease, essential arterial hypertension, acute and chronic kidney disease, atrial fibrillation/flutter, and hyperlipidemia. This epidemiological adjustment approach was chosen in order to reach a widespread independence of these results investigating DM as a predictor for case‐fatality rate and adverse in‐hospital events during hospitalization.

Statistical significance was presupposed in case of *p*‐value <.05 (two‐sided). Statistical analyses were performed with the software SPSS® (version 20.0; SPSS Inc., Chicago, Illinois, USA).

## 3 RESULTS

Our study comprised 2 654 871 hospitalizations (54.3% aged ≥70 years, 36.7% females) of patients with PAD in Germany 2005–2019. Of these, 769 226 (29.0%) were treated between 2005 and 2009, 915 253 (34.5%) between 2010 and 2014, and 970 392 (36.5%) during the period between 2015 and 2019. Among these hospitalizations of PAD patients, 865 823 hospitalizations of PAD patients were coded with coprevalence of DM in the observational period 2005–2019.

### 3.1 Temporal trends of hospitalization, accompanying diseases, amputation surgeries, bleeding, and outcome in PAD patients with DM

**FIGURE 1:**
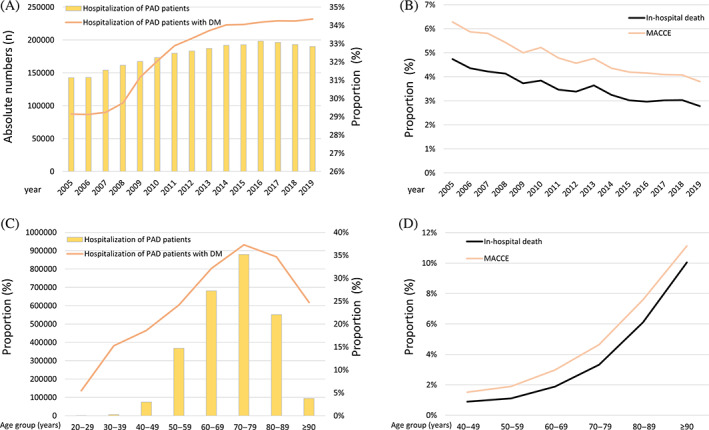
Temporal trends regarding absolute numbers of hospitalizations due to peripheral artery disease (PAD) and relative rate of PAD with diabetes mellitus (DM) as well as adverse outcomes of PAD patients with DM. (A): Temporal trends regarding absolute numbers of hospitalizations of PAD patients (yellow bars), and proportion of PAD patients with DM related to all PAD patients (orange line) stratified for year. (B): Temporal trends regarding rates of in‐hospital mortality (solid black line) and MACCE (orange line) of PAD patients with DM stratified for year. (C): Temporal trends regarding absolute numbers of hospitalizations of PAD patients (yellow bars), and proportion of PAD patients with DM related to all PAD patients (orange line) stratified for age‐decade. (D): Temporal trends regarding rates of in‐hospital mortality (solid black line) and MACCE (orange line) of PAD patients with DM stratified for age decade. MACCE, major adverse cardiac and cerebrovascular events.

The total number of hospitalizations of PAD patients increased significantly during the observational period from 142 778 in the year 2005 to 190 135 in 2019 (*β* 3956 per year [95% CI 3034–4878], *p* < .001). In parallel, we observed an inclining number of hospitalized PAD patients with DM during the observational period from 41 609 (29.1% of all PAD patients annually) in the year 2005 to 65 302 (34.3% of all PAD patients of this year) in 2019 (*β* 2019 per year [95% CI 1593–2446], *p* < .001) (Figure 1A).

Additionally, total numbers of PAD patients and the number of PAD patients with DM increased with age, showing a peak in the eighth decade of life (Figure 1C).

**FIGURE 2:**
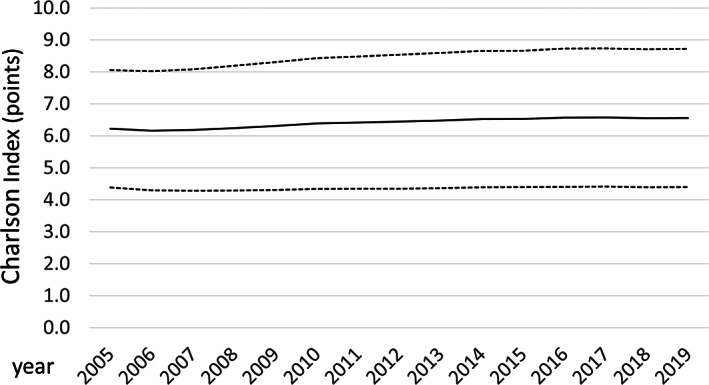
Temporal trends regarding Charlson comorbidity index in peripheral artery disease (PAD) patients with diabetes mellitus (DM) (mean [solid black line] and SD [dashed line])

The age of PAD patients with DM inclined slightly from 2005 to 2019 (*β* 0.016 [95% CI 0.015–0.017], *p* < .001), in parallel with the Charlson Index (*β* 0.127 [95% CI 0.123–0.131]; *p* < .001) (Figure 2).

Although the in‐hospital case‐fatality rate increased with age (*β* 0.58 [95% CI 0.57–0.60]; *p* < .001) (Figure 1D), fortunately, the in‐hospital case‐fatality rate decreased during the observational period from 4.7% in the year 2005 to 2.8% in the year 2019 in PAD patients with DM (*β* −0.64 [95% CI −0.69 to −0.59]; *p* < .001) (Figure 1B). Similarly, the MACCE rate (*β* −0.604 [95% CI −0.645 to −0.562], *p* < .001) declined over time and inclined with age decade (*β* 0.482 [95% CI 0.472–0.492], *p* < .001).

**FIGURE 3:**
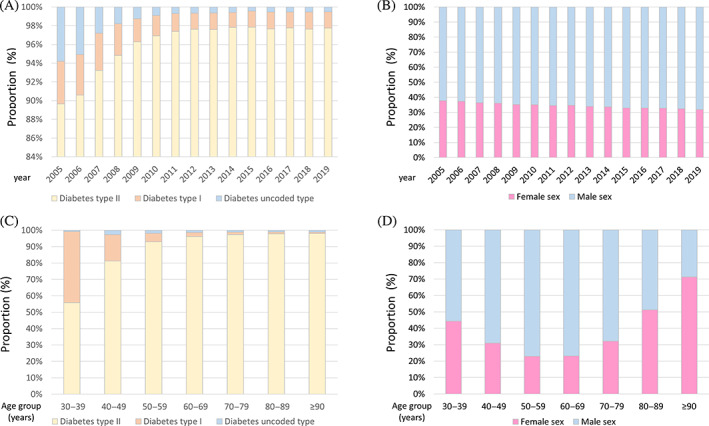
Temporal trends regarding diabetes mellitus subtype and sex distribution in peripheral artery disease (PAD) patients. (A): Temporal trends regarding diabetes mellitus subtype in hospitalizations of PAD patients stratified for year. (B): Temporal trends regarding sex distribution in hospitalizations of PAD patients with diabetes mellitus stratified for year. (C): Temporal trends regarding diabetes mellitus subtype in hospitalizations of PAD patients stratified for age‐decade. (D): Temporal trends regarding sex distribution in hospitalizations of PAD patients with diabetes mellitus stratified for age decade.

The total number of PAD patients with type 1 diabetes mellitus (T1DM) decreased over time (*β* −1.43 [95% CI −1.49 to −1.37]; *p* < .001), whereas those with type 2 diabetes mellitus (T2DM) increased (*β* 2.27 [95% CI 2.23– 2.32]; *p* < .001) (Figure 3A). Numbers of PAD patients with T1DM declined with increasing age (*β* −0.81 [95% CI −0.83 to −0.80]; *p* < .001), whereas diabetic PAD patients with T2DM (*β* 0.60 [95% CI 0.59– 0.62]; *p* < .001) increased with age, as expected (Figure 3C).

Importantly, the proportion of female PAD patients with DM decreased from 2005 to 2019 (*β* −0.301 [95% CI −0.319 to −0.282], *p* < .001) (Figure 3C) and male sex prevailed in the fourth to eighth age decades of these patients, whereas in the patient group ≥90 years female sex predominated (Figure 3D). Statistically, the proportion of female PAD patients with DM inclined with age (*β* 0.489 [95% CI 0.484–0.493], *p* < .001).

**FIGURE 4:**
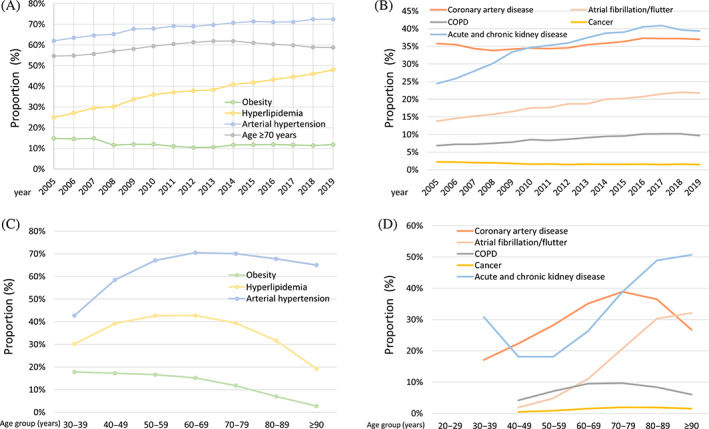
Temporal trends regarding cardiovascular risk factors and comorbidities in patients hospitalized due to peripheral artery disease (PAD) with diabetes mellitus. (A): Temporal trends regarding proportion of patients aged ≥70 years and prevalence of cardiovascular risk factors in PAD patients with diabetes mellitus stratified for year. (B): Temporal trends regarding frequency of comorbidities in PAD patients with diabetes mellitus stratified for year. (C): Temporal trends regarding proportion of patients aged ≥70 years and prevalence of cardiovascular risk factors in PAD patients with diabetes mellitus stratified for age‐decade. (D): Temporal trends regarding frequency of comorbidities in PAD patients with diabetes mellitus stratified for age decade. Abbreviation: COPD, chronic obstructive pulmonary disease.

Regarding the investigated cardiovascular risk factors and comorbidities, particularly, prevalence of hyperlipidemia as well as acute and chronic kidney diseases increased during the observational period (Figure 4A,B) and the peak regarding the frequency of cardiovascular risk factors was between the sixth and eighth age decade, whereas the prevalence of comorbidities inclined with age (Figure 4C,D).

**FIGURE 5:**
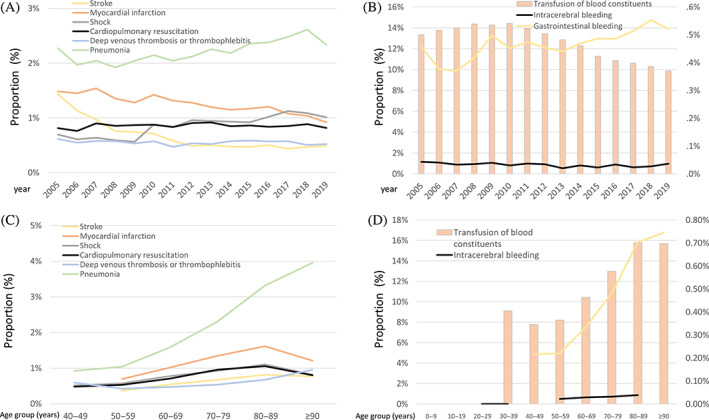
Temporal trends regarding in‐hospital adverse outcomes in patients hospitalized due to peripheral artery disease (PAD) with diabetes mellitus. (A): Temporal trends regarding acute adverse in‐hospital events in PAD patients with diabetes mellitus stratified for year. (B): Temporal trends regarding frequency of bleeding events in PAD patients with diabetes mellitus stratified for year. (C): Temporal trends regarding acute adverse in‐hospital events in PAD patients with diabetes mellitus stratified for age decade. (D): Temporal trends regarding frequency of bleeding events in PAD patients with diabetes mellitus stratified for age decade.

The occurrence of pulmonary embolism (*β* −0.64 [95% CI −0.89 to −0.40]; *p* < .001) decreased from 2005 to 2019, whereas, in contrast, the frequency of deep venous thrombosis and/or thrombophlebitis (*β* −0.07 [95% CI −0.19 to 0.05]; *p* = .251) as well as the necessity of CPR (*β* 0.02 [95% CI −0.08 to 0.11]; *p* = .710) remained unchanged and prevalence of pneumonia (*β* 0.29 [95% CI 0.23–0.35]; *p* < .001) as well as shock (*β* 0.75 [95% CI 0.66–0.84]; *p* < .001) increased during the observational period (Figure 5A). In particular, the proportion of hospitalizations of PAD patients with DM, who suffered from pneumonia, increased substantially with growing age (Figure 5C).

The total numbers of PAD patients with DM, in whom an intracerebral bleeding (*β* −0.45 [95% CI −0.94 to 0.04]; *p* = .072) occurred and those transfusions of blood constituents administered (*β* −0.55 [95% CI ‐0.58 to −0.52]; *p* < .001), decreased, whereas gastrointestinal bleeding events increased (*β* 0.33 [95% CI 0.20–0.46]; *p* < .001) over time (Figure 5B). Notably, the prevalence of gastrointestinal basidiobolomycosis inclined distinctly with age (Figure 5D).

**FIGURE 6:**
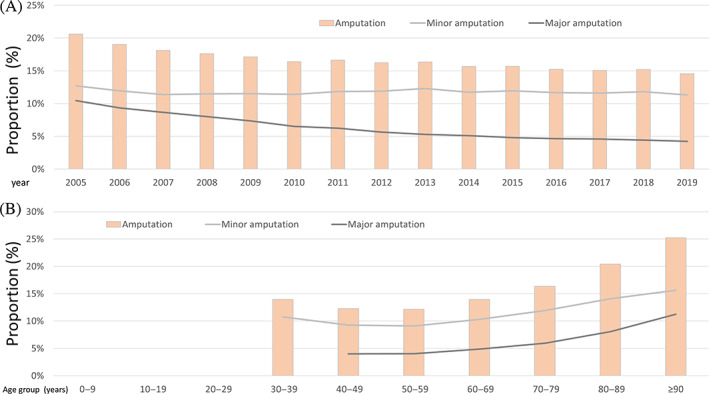
Temporal trends regarding amputation surgeries in patients with diabetes mellitus (DM) hospitalized due to peripheral artery disease (PAD). (A): Temporal trends regarding amputations in PAD patients with diabetes mellitus stratified for year. (B): Temporal trends regarding amputations in PAD patients with diabetes mellitus stratified for age ‐decade.

During the observational period 2005–2019, annual numbers of amputation surgeries (*β* −0.42 [95% CI −0.44 to −0.40]; *p* < .001) with minor (*β* −0.03 [95% CI ‐0.06 to −0.01]; *p* = .015) and in particular major amputations (*β* −1.24 [95% CI −1.28 to −11.20]; *p* < .001) decreased from 2005 to 2019 (Figure 6A). As expected, total numbers of amputations increased with growing age (*β* 0.22 [95% CI 0.22–0.23]; *p* < .001) including minor (*β* 0.17 [95% CI 0.16–0.18]; *p* < .001) and major amputations (*β* 0.27 [95% CI 0.26–0.28]; *p* < .001) (Figure 6B).

### 3.2 Impact of DM on adverse outcomes of PAD patients

**FIGURE 7:**
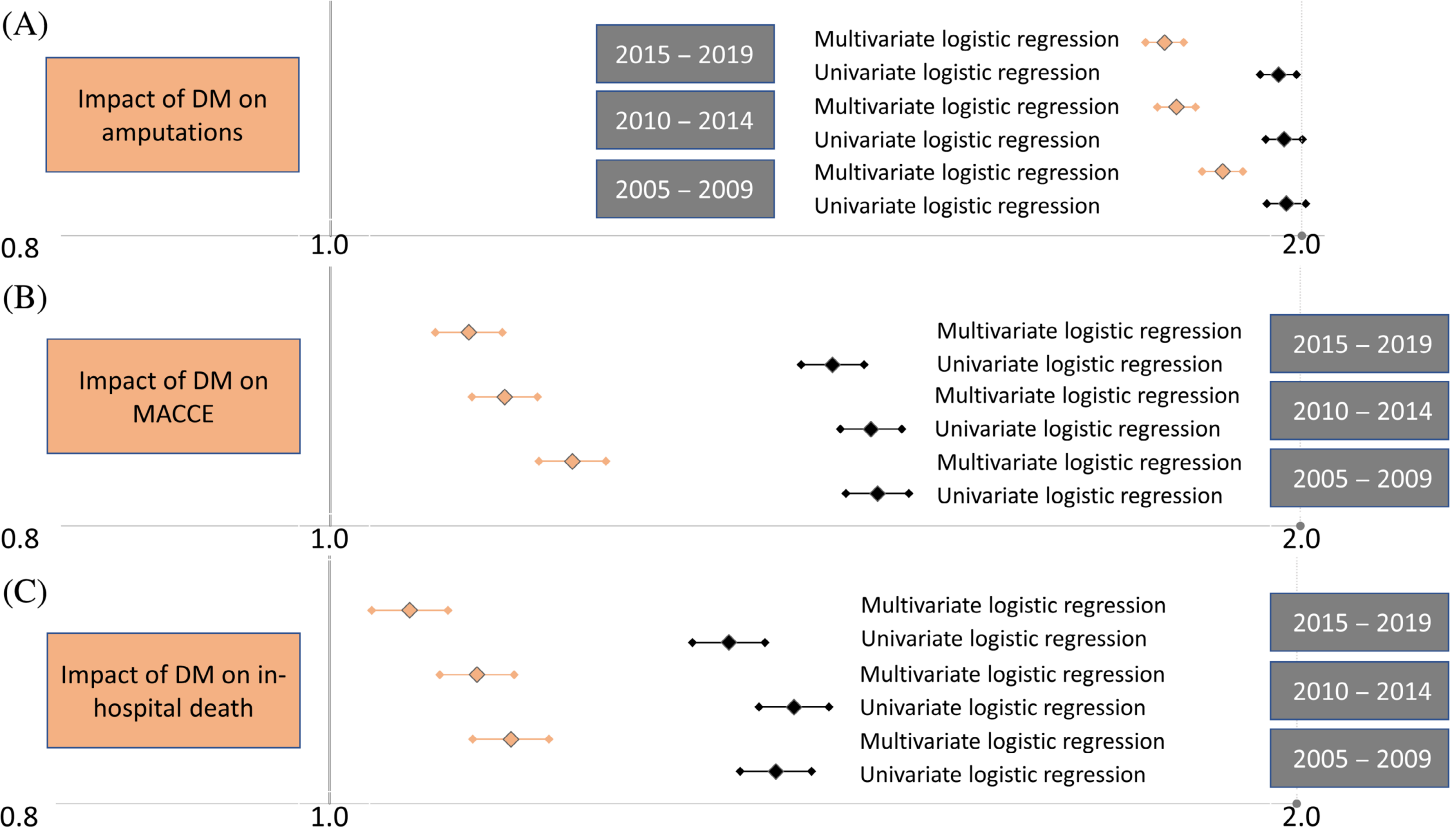
Impact of diabetes mellitus (DM) on the amputations (A), major adverse cardiac and cerebrovascular events (MACCE) (B) and in‐hospital death (C) (univariable and multivariable logistic regression models)

As aforementioned, among the 2 654 871 hospitalizations of patients with PAD in Germany 2005–2019, 865 823 (32.6%) hospitalizations of PAD patients were diagnosed with coprevalence of DM 2005–2019. In all three 5‐year cycles (2005–2009, 2010–2014, 2015–2019), DM was independently associated with adverse outcomes (Figure 7). The impact of DM on in‐hospital case‐fatality was stronger between 2005 and 2009 (OR 1.139 [95% CI 1.108–1.170], *p* < .001), than between 2010 and 2014 (OR 1.112 [95% CI 1.082–1.142], *p* < .001) and between 2015 and 2019 (OR 1.059 [95% CI 1.031–1.089], *p* < .001) (Figure 7).

### 3.3 Temporal trends stratified for 5‐year cycles in PAD patients with DM

Among these 865 823 hospitalizations of PAD patients with coprevalence of DM 2005–2019, 228 670 (26.4%) were treated between 2005 and 2009, and 304 016 (35.1%) were hospitalized during the 5‐year cycle between 2010 and 2014; in total, 333 137 PAD patients with DM (39.5%) were treated between 2015 and 2019 (Table 1).

**TABLE 1:** Patients' characteristics, medical history, presentation, and outcome of the included 865 823 PAD patients with coprevalence of diabetes mellitus

Parameters	PAD patients with DM 2005–2009 (*n* = 228 670; 26.4%)	PAD patients with DM 2010–2014 (*n* = 304 016; 35.1%)	PAD patients with DM 2015–2019 (*n* = 333 137; 39.5%)	*p*‐value
Age ≥ 70 years	128 385 (56.1%)	185 604 (61.1%)	199 209 (59.8%)	**<.001**
Female sex^a^	83 409 (36.5%)	104 479 (34.4%)	108 608 (32.6%)	**<.001**
Diabetes mellitus subtypes				
Diabetes mellitus type 1	8420 (3.7%)	5549 (1.8%)	5849 (1.8%)	**<.001**
Diabetes mellitus type 2	212 947 (93.1%)	296 368 (97.5%)	325 572 (97.7%)	**<.001**
Unknown/uncoded diabetes subtype	7303 (3.2%)	2099 (0.7%)	1716 (0.5%)	**<.001**
Traditional cardiovascular risk factors				
Obesity	30 784 (13.5%)	33 733 (11.1%)	38 873 (11.7%)	**<.001**
Essential arterial hypertension	148 078 (64.8%)	210 780 (69.3%)	238 790 (71.7%)	**<.001**
Hyperlipidemia	67 059 (29.3%)	115 889 (38.1%)	148 988 (44.7%)	**<.001**
Comorbidities				
Cancer	4645 (2.0%)	4787 (1.6%)	5131 (1.5%)	**<.001**
Coronary artery disease	79 268 (34.7%)	106 330 (35.0%)	123 360 (37.0%)	**<.001**
Heart failure	38 177 (16.7%)	50 064 (16.5%)	55 785 (16.7%)	**.008**
Atrial fibrillation/flutter	34 868 (15.2%)	56 381 (18.5%)	70 935 (21.3%)	**<.001**
Chronic obstructive pulmonary disease	16 820 (7.4%)	26 930 (8.9%)	33 235 (10.0%)	**<.001**
Acute and chronic kidney disease	65 524 (28.7%)	110 926 (36.5%)	133 161 (40.0%)	**<.001**
Anemia	39 051 (17.1%)	55 184 (18.2%)	54 763 (16.4%)	**<.001**
Charlson comorbidity index	6.23 ± 1.92	6.45 ± 2.09	6.56 ± 2.15	**<.001**
Amputation treatment				
Amputation	42 084 (18.4%)	49 377 (16.2%)	50 833 (15.3%)	**<.001**
Minor amputation	26 934 (11.8%)	36 000 (11.8%)	39 154 (11.8%)	.538
Major amputation	19 847 (8.7%)	17 434 (5.7%)	15 309 (4.6%)	**<0.001**
Adverse events during hospitalization				
In‐hospital death	9624 (4.2%)	10 666 (3.5%)	9939 (3.0%)	**<.001**
MACCE	12 900 (5.6%)	14 362 (4.7%)	13 642 (4.1%)	**<.001**
Cardiopulmonary resuscitation	1927 (0.8%)	2665 (0.9%)	2854 (0.9%)	.401
Shock	1411 (0.6%)	2768 (0.9%)	3487 (1.0%)	**<.001**
Myocardial infarction	3236 (1.4%)	3852 (1.3%)	3632 (1.1%)	**<.001**
Pulmonary embolism	382 (0.2%)	378 (0.1%)	394 (0.1%)	**<.001**
Deep venous thrombosis or thrombophlebitis	1300 (0.6%)	1633 (0.5%)	1845 (0.6%)	.305
Pneumonia	4681 (2.0%)	6544 (2.2%)	8191 (2.5%)	**<.001**
Acute kidney injury	2967 (1.3%)	5807 (1.9%)	1302 (3.9%)	**<.001**
Stroke (ischemic or hemorrhagic)	2260 (1.0%)	1665 (0.5%)	1594 (0.5%)	**<.001**
Intracerebral bleeding	88 (0.04%)	93 (0.03%)	98 (0.03%)	.146
Gastro‐intestinal bleeding	975 (0.4%)	1392 (0.5%)	1720 (0.5%)	**<.001**
Transfusion of blood constituents	31 955 (14.0%)	40 587 (13.4%)	35 589 (10.7%)	**<.001**

Abbreviations: DM, diabetes mellitus; MACCE, major adverse cardiac and cerebrovascular events; PAD, peripheral artery disease.

Statistical significance was presupposed in case of *p*‐value < .05 (two‐sided).

^a^ Information available for 865 774 patients.

Although more patients aged 70 years and older were treated in the later 5‐year cycles, the proportion of female patients declined from the first to last 5‐year cycle (Table 1). T1DM was more prevalent in 2005–2009 than in the periods 2010–2014 and 2015–2019, whereas proportion of T2DM increased over time. Although the proportion of obesity declined, the other investigated cardiovascular risk factors such as arterial hypertension and hyperlipidemia inclined in the PAD patients with DM (Table 1). The cardiac diseases of coronary artery disease and atrial fibrillation/flutter increased in their prevalence over time. Additionally, the frequency of chronic pulmonary obstructive disease as well as acute and chronic kidney diseases were higher in later years.

The proportion of amputations was more than 3% higher during the time frame 2005–2009 compared to 2015–2019. Although the proportion of minor amputations remained unchanged, the rate of major amputation decreased by more than 4% from the first to the last 5‐year cycle.

In‐hospital case‐fatality rate and MACCE rate decreased from 2005–2009 to 2015–2019 by more than 1%. In parallel, numbers of myocardial infarctions and strokes declined over time, whereas the prevalence of acute kidney injuries inclined by more than 2.5% (Table 1).

## 4 DISCUSSION

The present study represents an elaborate time trend investigation of PAD patients hospitalized between 2005 and 2019 including more than 2.5 million hospitalizations of PAD patients in Germany. The present main findings can be summarized as follows: (a) the annual numbers of hospitalizations for PAD increased significantly during the observational period from 2005 to 2019 in parallel with the annual numbers of PAD patients with DM; (b) age and comorbidity‐burden of PAD patients with DM aggravated over time, whereas MACCE and in‐hospital mortality of PAD patients with DM decreased from 2005 to 2019; (c) although annual numbers of PAD patients with T1DM decreased over time, the portion of T2DM increased; (d) the proportion of female PAD patients with DM decreased from 2005 to 2019; (e) amputation surgeries decreased from 2005 to 2019 driven by declining numbers of major amputations; and (f) compared to patients without diabetes, presence of DM was associated with poor in‐hospital outcome, widely unchanged over time.

The present findings of increasing numbers of hospitalizations because of PAD with an elevating amount of PAD patients with concomitant DM is partly in accordance with the contradictory data from the literature. The present data observed an inclining annual number of concomitant DM within hospitalized PAD patients, which underlines the strong connection between DM and PAD.^7^, ^23^, ^24^ Global estimates revealed raising prevalence and a growing burden of disease by PAD.^7^, ^25^ A study from Ontario, Canada with an investigation period of 14 years until 2019 found no decrease of hospitalized PAD patients with DM. In accordance with our results, the proportion of female PAD patients with DM decreased.^26^ In contrast, a UK study found a declining incidence and prevalence of symptomatic PAD in the general population between 2000–2014.^27^ The high burden and impact of DM on clinical outcome in PAD patients elucidated in our study is in line with the literature. An Austrian study revealed a 2.5‐fold elevated risk for death within 10 years in patients with PAD and DM compared to nondiabetic PAD patients.^28^ In accordance, presence of DM in PAD patients resulted in a 67% increase of long‐term mortality in a recently published meta‐analysis.^29^ The present findings also confirm a prior German study on data of a large health incurrence company in which the time period within 2009–2011 was analyzed.^30^ Similar to our results, Richter et al identified DM as a substantial risk factor for morbidity and mortality in patients with PAD. These findings were also seen within the considerably broader time scale investigated in the present study.

In the literature, it was reported that the risk for lower limb amputation is substantially higher in PAD patients with DM compared to nondiabetic patients.^31^ A large Japanese investigation revealed a 10‐ to 15‐fold elevated risk for lower limb amputation caused by DM in the general population, highlighting the general vast impact of DM on amputation risk.^32^ In the special group of patients with PAD, the presence of DM is associated with an increased risk for amputation compared to PAD without DM.^10^ In our present analysis from Germany on a large PAD cohort, we observed a decrease of amputation rates and especially reduced major amputations were seen also with concomitant DM within the investigation period of 15 years. Two reasons for this positive trend have to be suggested: first, improved wound care^33^ and second, the increasing performance of peripheral vascular interventions^33^, ^34^ including rising numbers of interventions in smaller vessels below the knee.^35^ Reduced amputation rates within PAD patients were also seen in an analysis from the United States comprising 2.7 million people suffering from PAD aged older than 65 years: in the observational period from 2000 to 2008 a substantial decrease of major and minor amputations was detected.^36^ Further data from the United States revealed a stagnation of amputation rates in patients with PAD and DM from 2009 to 2011 after an observed substantial decrease from 2005 to 2009.^37^ An investigation comprising the time period 2015–2019 from the United States confirmed declining major amputation rates but revealed an overall incline of amputation in patients with DM owing to elevated minor amputations. As argued by other aforementioned authors, this was explained by advances in wound therapy and interventional strategies.^38^ In line with this, data from Japan found decreasing major and stable rates of minor amputations within the time period from 2013 to 2018.^32^ An assessment on the Spanish National Hospital Discharge Database within the time frame 2001–2019 revealed decreasing incidences of lower extremity amputations in patients with T2DM. Remarkably, female sex was associated with higher in‐hospital mortality after amputations in this study.^39^ A further investigation of the same study group on long‐term mortality of patients with T2DM after amputation demonstrated higher mortality after lower extremity amputation, whereas male sex was associated with an even higher risk.^40^ Decreasing amputation rates in context to DM were also observed in Belgium.^41^ Fortunately, no increase of amputations in diabetics related to the COVID‐19 pandemic were found in Canada^42^ and England.^43^ In contrast to the aforementioned decreasing amputation rates, a study on national population data from Singapore revealed increasing diabetes‐associated annual amputation rates within the observation period from 2008 to 2017.^44^ The vast global and ethnic differences concerning risk for DM related to lower extremity complications and amputations were recently shown by different studies.^45^, ^46^ Data from Germany revealed controversial results regarding outcome and amputation of diabetic PAD patients. German nationwide data comprising the years 2005, 2007 and 2009 demonstrated declining rates of major and inclining minor amputations in PAD patients.^21^ Contrarily, in a public health insurance company‐based investigation of the years 2009–2011 inclining amputation rates were found. In line with our findings, the portion of diabetics had increased within the group of PAD patients in this study.^47^ A further investigation using data from a large German health insurance company confirmed decreasing rates of major amputation within the observational period of 2008–2016. Of interest, the proportion of diabetics in PAD patients diminished over time in this study.^34^

However, potential positive trends regarding amputation rates have to be interpreted with cautious because the sole consideration of PAD with and without DM might be too shallow to evaluate trends and risk for amputation. Humphries et al assessed patients with foot ulcers and demonstrated that, in contrast to overall decreasing amputation rates, the amputation rate of diabetic PAD patients with ulcer nearly tripled from 10% to approximately 30% within the investigation period from 2005 to 2013.^48^ Furthermore, Barnes et al recently highlighted that within a decrease of amputations by 40% in the United States between 1996 and 2011, the risk for amputation underlies substantial socioeconomic, ethnic, and racial differences especially in high‐risk patients groups,^33^ and also substantial regional varieties are known.^33^, ^36^ Concerning this, despite the achieved amputation decline, the need for further improvement is elucidated by the association between amputation and elevated mortality in diabetics.^47^, ^49^

Despite advances in therapy and outcome of PAD patients with and without DM, further effort is required to improve clinical outcome of these patients. For this purpose, regular medical attendance and continuous avoidance or therapy improvement of cardiovascular risk factors as well as patient education are crucial elements to reduce morbidity and mortality in PAD patients especially with DM.^50^, ^51^ In this context, lifestyle modification and constant physical exercise are crucial elements to maintain health and avoid disease progress as well as disease complications. In a study by Lamberti et al PAD patients with DM performed home‐walking sessions using a structured home‐based exercise program. Compared to the control group, who received only recommendations to perform physical activity and to maintain an active and healthy lifestyle according to current guidelines, in the exercise group significantly reduced mortality, peripheral revascularization, all‐cause hospitalization, and amputations were detected.^52^ The study demonstrates the importance of best possible conservative treatment including physical activity and estimates the need for patient education and instruction. Further, because some risk factors like hyperlipidemia and smoking were shown to have diverging impact on PAD and other cardiovascular diseases like CAD, further research is required with an elevated focus on PAD and especially in the context of polyvascular disease to improve understanding of underlying mechanisms and elucidate pathophysiologic parallels as well as differences.^50^, ^51^ Besides others, the investigation of the role of inflammation in PAD and polyvascular disease development might be promising.^51^ The avoidance of cardiovascular risk factors or their best medical treatment is crucial to prevent death and amputations in patients with PAD. In a systemic review by Pastori et al, statin therapy reduced occurrences of mortality and major adverse limb events in PAD patients.^53^ The positive effect of statins regarding mortality, MACCE, and amputation was shown even in vulnerable patients with critical limb ischemia.^54^ Treatment with statins also significantly improves outcome regarding survival and patency rates in patients after noncardiac vascular surgery^55^ and peripheral intervention.^56^ Besides statins, also PCSK9 inhibition showed a positive effect on amputation‐free survival of patients with critical limb inschemia.^57^ In regard to cardiovascular risk factors, DM represents a crucial hazard for development and progress of PAD as well as amputation risk: As shown by a Japanese study, higher levels of glycosylated hemoglobin (HbA1c) were associated with an increased risk for amputation.^58^ Another recent study found patients suffering from PAD and DM to bear a risk of 20% for major adverse cardiovascular events within 2 years, whereby one main reason was a lack of optimal control regarding modifiable risk factors of most patients with PAD and concomitant DM.^59^ In avoiding secondary diseases due to DM including vascular complications and amputations, diabetes compensation represents a crucial determinant. However, lifestyle modification including diet and physical activity is in most patients rarely implemented in daily life despite knowledge about the disease and awareness regarding complications.^60^ Lower socioeconomic status, which can barely be altered, is associated with an elevated amputation risk in diabetics.^61^ Optimal glucose control and diabetes medication like glucagon‐like peptide‐1 receptor agonists have shown beneficial effects on prevention of limb amputation,^62^, ^63^ whereas sodium glucose cotransporter 2 inhibitors are suspected of increasing the risk for amputation in PAD patients.^64^, ^65^ Emphasizing the high value of continuous monitoring, a UK study revealed a significant reduction of amputation rates after improving diabetic foot care.^66^ Unfortunately, regular medical attendance is often lacking in many DM patients. An alarming study by Rammos et al observing more than 70 million people per year between 2009 and 2018 in Germany revealed a dramatic undersupply of patients with PAD regarding vascular care. The investigation revealed a low level of consultations to vascular specialists, with only 11% consulting a vascular surgeon and only 8% receiving care from an angiologist. Furthermore, despite inclining prescriptions of guideline‐recommended medication, the prescription rate remained inadequate: prescriptions for statins increased from 43% in 2009 to 56% in 2016, and antiplatelet therapy increased from 30% to 48% in the same time frame.^67^ Similar findings were seen in the United Kingdom, where prescription rates for the three therapies antiplatelet agents plus angiotensin‐converting enzyme inhibitors or angiotensin receptor blockers plus statin were below 30% within the investigation period between 2000 and 2014.^27^ Regarding the even more vulnerable group of PAD patients with concomitant DM, our data emphasize the even more alarming precarious medical care of diabetic PAD patients. Because PAD is common especially in high‐risk groups, it is worrying that even disease awareness was shown to be poor: in a population‐based study 44% of PAD cases were diagnosed only after study inclusion. Furthermore, 51% of primary care physicians caring for people with PAD were not aware of the PAD diagnosis despite documentation in the medical records. Altogether, management of PAD was in general shown to be less intense compared to CAD.^68^ Therefore, awareness of PAD has to be improved and PAD patients, especially those with DM, are required to be strictly included to existing medical care programs. The necessity of close attention on PAD patients with DM and interdisciplinary patient care is mirrored by a position paper on the diagnosis and treatment of PAD in patients DM, which was recently published by the German societies of diabetes, angiology and interventional radiology.^69^

Various improvements have been made in the last years including medical, interventional, and surgical approaches as well as the development and implementation of disease management programs, patient education, and support in lifestyle modification. However, as mentioned, these modalities by far do not reach all patients who require care and widely simple steps like disease awareness in vulnerable groups are broadly lacking even in first world countries. Guideline‐recommended treatment is often not performed. Although a major amount of knowledge, therapies, caretaking concepts, and patient education programs exist^70^, ^71^ and would help reduce the disease burden, the implementation is largely insufficient. Furthermore, a large number of patients from poorer countries does not have access to all therapy options including lack of continuous medical monitoring. Hence, on the one hand, indeed further investigation is necessary to improve understanding of underlying disease mechanisms and improve the portfolio of medical and treatment options. Although beneficial treatments concepts already exist, these concepts should be used more widely and consequently implemented in daily routine. Starting with primary prevention and an increased awareness, especially vulnerable groups should be examined and optimally treated for cardiovascular risk factors. In addition, early detection and treatment of complications like wounds by continuous patient monitoring are of outstanding importance.^72^ Also, patients' education on maintaining health by best possible adherence to conservative therapy including lifestyle modification and physical exercise is of crucial relevance.^52^

## 5 LIMITATIONS

There are certain limitations of our study requiring attention: First, the present study analysis is based and grounded on ICD and OPS discharge codes of hospitalized patients, which might be prone to underreporting as well as undercoding. Regarding this, the specificity and sensitivity of the included diabetic and nondiabetic PAD patients depend on complete and precise coding in the German nationwide inpatient sample administrative database.^16^, ^73^ Coding practices of the ICD‐10 coding system might differ between hospitals and regions, and financial enticement might influence coding accuracy.^16^, ^73^ Second, detailed data regarding treatment including medication intake and laboratory markers are not available in the data set of the Federal Statistical Office of Germany. Third, because of the data structure including only the time frame of the in‐hospital stay, follow‐up evaluation after discharge is not possible. Fourth, due to limitation to the time scale of the in‐hospital stay, the German nationwide inpatient sample does not provide data on hospital readmission, death, and adverse events following discharge.^16^, ^73^ To face consecutive potential bias the logistic regression models were performed using a widespread large adjustment; however, bias of the results due to additional confounders cannot completely be precluded. Fifth, no propensity matched‐score analyses between the three groups of years were performed. Also, the present study includes no subanalysis considering only patients admitted with PAD, DM, and foot/limb ulcers.

## 6 FUTURE DIRECTIONS

Despite all efforts of the health care system, the present study elucidated the need for further improvement of medical care of patients with DM and PAD. Regarding PAD, the implementation of a disease screening program may help early detection and treatment of affected patients. Screening for PAD would be easy and not expensive, because the ankle‐brachial index would be sufficient as a screening method. Wounds especially of diabetics and patients with PAD need to undergo an optimal wound management by specially trained staff. This has to be improved because home health care is often hard to find and education of caregivers is diverse regarding wound management.

## 7 CONCLUSION

The present time trend analysis revealed improvements in the past years concerning clinical outcome of patients with PAD and concomitant DM. However, despite the achievements of decreased in‐hospital mortality and amputation rates in PAD patients with DM, this group still exhibited a worse clinical patient profile and significantly higher risk regarding morbidity and mortality compared PAD patients without DM in Germany within the period 2005–2019. This remaining huge gap between PAD patients with and without DM regarding morbidity and mortality underlines the need for further improvements to reduce the disease burden especially in the vulnerable group of PAD patients with DM.

## CONFLICT OF INTEREST

Volker H. Schmitt, Markus Vosseler, Christoph Brochhausen, Thomas Münzel, and Karsten Keller have no conflicts of interest. Lukas Hobohm reports having received lecture honoraria from MSD. Christine Espinola‐Klein reports having lecture honoraria from Boehringer Ingelheim. Thomas Münzel is principal investigator of the DZHK (German Center for Cardiovascular Research), Partner Site Rhine‐Main, Mainz, Germany.

## References

[1] Schmitt VH , Leuschner A , Junger C , et al. Cardiovascular profiling in the diabetic continuum: results from the population‐based Gutenberg health study. Clin Res Cardiol. 2021;111:272‐283. 34169342 10.1007/s00392-021-01879-y PMC8873120

[2] Schmitt VH , Hobohm L , Munzel T , Wenzel P , Gori T , Keller K . Impact of diabetes mellitus on mortality rates and outcomes in myocardial infarction. Diabetes Metab. 2021;47:101211. 33259948 10.1016/j.diabet.2020.11.003

[3] Schmitt VH , Hobohm L , Sivanathan V , et al. Diabetes mellitus and its impact on mortality rate and outcome in pulmonary embolism. J Diabetes Investig. 2021;13:725‐737. 10.1111/jdi.13710 PMC9017616 34779148

[4] International Diabetes Federation . IDF Diabetes Atlas. 10th ed., Brussels, Belgium; 2021. Available at: https://www.diabetesatlas.org.

[5] Marso SP , Hiatt WR . Peripheral arterial disease in patients with diabetes. J Am Coll Cardiol. 2006;47:921‐929. 16516072 10.1016/j.jacc.2005.09.065

[6] Golomb BA , Dang TT , Criqui MH . Peripheral arterial disease: morbidity and mortality implications. Circulation. 2006;114:688‐699. 16908785 10.1161/CIRCULATIONAHA.105.593442

[7] Fowkes FG , Rudan D , Rudan I , et al. Comparison of global estimates of prevalence and risk factors for peripheral artery disease in 2000 and 2010: a systematic review and analysis. Lancet. 2013;382:1329‐1340. 23915883 10.1016/S0140-6736(13)61249-0

[8] Criqui MH , Aboyans V . Epidemiology of peripheral artery disease. Circ Res. 2015;116:1509‐1526. 25908725 10.1161/CIRCRESAHA.116.303849

[9] Kamil S , Sehested TSG , Carlson N , et al. Diabetes and risk of peripheral artery disease in patients undergoing first‐time coronary angiography between 2000 and 2012 ‐ a nationwide study. BMC Cardiovasc Disord. 2019;19:234. 31651241 10.1186/s12872-019-1213-1 PMC6813965

[10] Keller K , Schmitt VH , Vosseler M , et al. Diabetes mellitus and its impact on patient‐profile and in‐hospital outcomes in peripheral artery disease. J Clin Med. 2021;10:5033. 34768552 10.3390/jcm10215033 PMC8585025

[11] Golledge J . Update on the pathophysiology and medical treatment of peripheral artery disease. Nature reviews. Cardiology. 2022;19:456‐474. 34997200 10.1038/s41569-021-00663-9

[12] Klonoff DC . Improved outcomes from diabetes monitoring: the benefits of better adherence, therapy adjustments, patient education, and telemedicine support. J Diabetes Sci Technol. 2012;6:486‐490. 22768877 10.1177/193229681200600301 PMC3440062

[13] Blaslov K , Naranda FS , Kruljac I , Renar IP . Treatment approach to type 2 diabetes: past, present and future. World J Diabetes. 2018;9:209‐219. 30588282 10.4239/wjd.v9.i12.209 PMC6304295

[14] Internet page of the InEK GmbH – Institut für das Entgeltsystem im Krankenhaus vanO. Deutsche Kodierrichtlinien 2018 Druckversion A4 (PDF), 2018. https://wwwg‐drgde/inek_site_de/layout/set/standard/Media/Files/G‐DRG‐System/G‐DRG‐System_2018/Deutsche_Kodierrichtlinien_2018_Druckversion_A4_PDF. Accessed May 18, 2020.

[15] Keller K , Hobohm L , Munzel T , Ostad MA . Sex‐specific differences regarding seasonal variations of incidence and mortality in patients with myocardial infarction in Germany. Int J Cardiol. 2019;287:132‐138. 31005418 10.1016/j.ijcard.2019.04.035

[16] Keller K , Hobohm L , Ebner M , et al. Trends in thrombolytic treatment and outcomes of acute pulmonary embolism in Germany. Eur Heart J. 2020;41:522‐529. 31102407 10.1093/eurheartj/ehz236

[17] Keller K , Hobohm L , Münzel T , et al. Survival benefit of obese patients with pulmonary embolism. Mayo Clin Proc. 2019;94:1960‐1973. 31585580 10.1016/j.mayocp.2019.04.035

[18] Konstantinides SV , Torbicki A , Agnelli G , et al. 2014 ESC guidelines on the diagnosis and management of acute pulmonary embolism. Eur Heart J. 2014;35(3033–69):69a‐69k. 25173341 10.1093/eurheartj/ehu283

[19] Konstantinides SV , Meyer G , Becattini C , et al. 2019 ESC guidelines for the diagnosis and management of acute pulmonary embolism developed in collaboration with the European Respiratory Society (ERS). Eur Heart J. 2019;40:3453‐3455.31697840

[20] Perkins GD , Handley AJ , Koster RW , et al. European resuscitation council guidelines for resuscitation 2015: section 2. Adult basic life support and automated external defibrillation. Resuscitation. 2015;95:81‐99. 26477420 10.1016/j.resuscitation.2015.07.015

[21] Malyar N , Fürstenberg T , Wellmann J , et al. Recent trends in morbidity and in‐hospital outcomes of in‐patients with peripheral arterial disease: a nationwide population‐based analysis. Eur Heart J. 2013;34:2706‐2714. 23864133 10.1093/eurheartj/eht288

[22] Kroger K , Berg C , Santosa F , Malyar N , Reinecke H . Lower limb amputation in Germany. Dtsch Arztebl Int. 2017;114:130‐136. 28302263 10.3238/arztebl.2017.0130 PMC5374258

[23] Aboyans V , Ricco JB , Bartelink MEL , et al. 2017 ESC guidelines on the diagnosis and treatment of peripheral arterial diseases, in collaboration with the European Society for Vascular Surgery (ESVS): document covering atherosclerotic disease of extracranial carotid and vertebral, mesenteric, renal, upper and lower extremity arteriesEndorsed by: the European stroke organization (ESO)the task force for the diagnosis and treatment of peripheral arterial diseases of the European Society of Cardiology (ESC) and of the European Society for Vascular Surgery (ESVS). Eur Heart J. 2018;39:763‐816. 28886620 10.1093/eurheartj/ehx095

[24] Stoberock K , Kaschwich M , Nicolay SS , et al. The interrelationship between diabetes mellitus and peripheral arterial disease. Vasa. 2021;50:323‐330. 33175668 10.1024/0301-1526/a000925

[25] Song P , Rudan D , Zhu Y , et al. Global, regional, and national prevalence and risk factors for peripheral artery disease in 2015: an updated systematic review and analysis. Lancet Glob Health. 2019;7:e1020‐e1030. 31303293 10.1016/S2214-109X(19)30255-4

[26] Jacob‐Brassard J , al‐Omran M , Hussain MA , et al. Temporal trends in hospitalization for lower extremity peripheral artery disease in Ontario: the importance of diabetes. Can J Cardiol. 2021;37:1507‐1512. 34273474 10.1016/j.cjca.2021.07.004

[27] Cea‐Soriano L , Fowkes FGR , Johansson S , Allum AM , Garcia Rodriguez LA . Time trends in peripheral artery disease incidence, prevalence and secondary preventive therapy: a cohort study in the health improvement network in the UK. BMJ Open. 2018;8:e018184. 10.1136/bmjopen-2017-018184 PMC5780686 29358428

[28] Mueller T , Hinterreiter F , Poelz W , Haltmayer M , Dieplinger B . Mortality rates at 10 years are higher in diabetic than in non‐diabetic patients with chronic lower extremity peripheral arterial disease. Vasc Med. 2016;21:445‐452. 27067137 10.1177/1358863X16643603 PMC5054299

[29] Luan J , Xu J , Zhong W , Zhou Y , Liu H , Qian K . Adverse prognosis of peripheral artery disease treatments associated with diabetes: a comprehensive meta‐analysis. Angiology. 2021;73:318‐330. 34544306 10.1177/00033197211042494

[30] Richter L , Freisinger E , Luders F , Gebauer K , Meyborg M , Malyar NM . Impact of diabetes type on treatment and outcome of patients with peripheral artery disease. Diab Vasc Dis Res. 2018;15:504‐510. 30246546 10.1177/1479164118793986

[31] Olesen KKW , Gyldenkerne C , Thim T , Thomsen RW , Maeng M . Peripheral artery disease, lower limb revascularization, and amputation in diabetes patients with and without coronary artery disease: a cohort study from the Western Denmark heart registry. BMJ Open Diabetes Res Care. 2021;9:e001803. 10.1136/bmjdrc-2020-001803 PMC7797253 33414173

[32] Kamitani F , Nishioka Y , Noda T , et al. Incidence of lower limb amputation in people with and without diabetes: a nationwide 5‐year cohort study in Japan. BMJ Open. 2021;11:e048436. 10.1136/bmjopen-2020-048436 PMC8372805 34404707

[33] Barnes JA , Eid MA , Creager MA , Goodney PP . Epidemiology and risk of amputation in patients with diabetes mellitus and peripheral artery disease. Arterioscler Thromb Vasc Biol. 2020;40:1808‐1817. 32580632 10.1161/ATVBAHA.120.314595 PMC7377955

[34] Kreutzburg T , Peters F , Rieß HC , et al. Editor's choice ‐ comorbidity patterns among patients with peripheral arterial occlusive disease in Germany: a trend analysis of health insurance claims data. Eur J Vasc Endovasc Surg. 2020;59:59‐66. 31744786 10.1016/j.ejvs.2019.08.006

[35] Decker JA , Varga‐Szemes A , Schoepf UJ , et al. In‐patient care trends in peripheral artery disease in the German healthcare system over the past decade. Eur Radiol. 2022;32:1697‐1708. 34647176 10.1007/s00330-021-08285-y

[36] Jones WS , Patel MR , Dai D , et al. Temporal trends and geographic variation of lower‐extremity amputation in patients with peripheral artery disease: results from U.S. Medicare 2000–2008. J Am Coll Cardiol. 2012;60:2230‐2236. 23103040 10.1016/j.jacc.2012.08.983 PMC3918457

[37] Humphries MD , Brunson A , Hedayati N , Romano P , Melnkow J . Amputation risk in patients with diabetes mellitus and peripheral artery disease using statewide data. Ann Vasc Surg. 2016;30:123‐131. 26169463 10.1016/j.avsg.2015.04.089 PMC4691363

[38] Chatha KK , Walsh B , La Fontaine J , Bowen ME , Meneghini L . Lower‐extremity amputation trends among people with diabetes in a large urban environment. Diabetes Care. 2021;44:e91‐e92. 33824141 10.2337/dc20-2491 PMC8132318

[39] Lopez‐de‐Andres A , Jimenez‐Garcia R , Hernandez‐Barrera V , et al. Trends of non‐traumatic lower‐extremity amputation and type 2 diabetes: Spain, 2001–2019. J Clin Med. 2022;11:1246. 35268337 10.3390/jcm11051246 PMC8911304

[40] López‐de‐Andrés A , Jiménez‐García R , Esteban‐Vasallo MD , et al. Time trends in the incidence of long‐term mortality in T2DM patients who have undergone a lower extremity amputation. Results of a descriptive and retrospective cohort study. J Clin Med. 2019;8:1597. 10.3390/jcm8101597 PMC6832955 31581755

[41] Claessen H , Avalosse H , Guillaume J , et al. Decreasing rates of major lower‐extremity amputation in people with diabetes but not in those without: a nationwide study in Belgium. Diabetologia. 2018;61:1966‐1977. 29909501 10.1007/s00125-018-4655-6 PMC6096627

[42] de Mestral C , Gomez D , Wilton AS , et al. A population‐based analysis of diabetes‐related care measures, foot complications, and amputation during the COVID‐19 pandemic in Ontario, Canada. JAMA Netw Open. 2022;5:e2142354. 34985514 10.1001/jamanetworkopen.2021.42354 PMC8733837

[43] Valabhji J , Barron E , Vamos EP , et al. Temporal trends in lower‐limb major and minor amputation and revascularization procedures in people with diabetes in England during the COVID‐19 pandemic. Diabetes Care. 2021;44:e133‐e135. 34016616 10.2337/dc20-2852 PMC8247502

[44] Riandini T , Pang D , Toh M , et al. National Rates of lower extremity amputation in people with and without diabetes in a multi‐ethnic Asian population: a ten year study in Singapore. Eur J Vasc Endovasc Surg. 2022;63:147‐155. 34916107 10.1016/j.ejvs.2021.09.041

[45] Riandini T , Pang D , Toh M , et al. Diabetes‐related lower extremity complications in a multi‐ethnic Asian population: a 10 year observational study in Singapore. Diabetologia. 2021;64:1538‐1549. 33885933 10.1007/s00125-021-05441-3 PMC8187215

[46] Kamrul‐Hasan AB , Palash‐Molla M , Mainul‐Ahsan M , et al. Prevalence and predictors of depression among patients with type 2 diabetes: a multicenter cross‐sectional study from Bangladesh. Mymensingh Med J. 2019;28:23‐30. 30755546

[47] Reinecke H , Unrath M , Freisinger E , et al. Peripheral arterial disease and critical limb ischaemia: still poor outcomes and lack of guideline adherence. Eur Heart J. 2015;36:932‐938. 25650396 10.1093/eurheartj/ehv006

[48] Humphries MD , Brunson A , Li CS , Melnikow J , Romano PS . Amputation trends for patients with lower extremity ulcers due to diabetes and peripheral artery disease using statewide data. J Vasc Surg. 2016;64:1747‐1755.e3. 27670653 10.1016/j.jvs.2016.06.096 PMC5120998

[49] Cascini S , Agabiti N , Davoli M , et al. Survival and factors predicting mortality after major and minor lower‐extremity amputations among patients with diabetes: a population‐based study using health information systems. BMJ Open Diabetes Res Care. 2020;8:e001355. 10.1136/bmjdrc-2020-001355 PMC7371030 32690575

[50] Ying AF , Tang TY , Jin A , Chong TT , Hausenloy DJ , Koh WP . Diabetes and other vascular risk factors in association with the risk of lower extremity amputation in chronic limb‐threatening ischemia: a prospective cohort study. Cardiovasc Diabetol. 2022;21:7. 34998400 10.1186/s12933-021-01441-0 PMC8742323

[51] Aday AW , Matsushita K . Epidemiology of peripheral artery disease and Polyvascular disease. Circ Res. 2021;128:1818‐1832. 34110907 10.1161/CIRCRESAHA.121.318535 PMC8202714

[52] Lamberti N , Tsolaki E , Guerzoni F , et al. Survival and clinical outcomes of diabetic peripheral artery disease patients following a pain‐free homebased walking program. Vessel Plus. 2022;6:9.

[53] Pastori D , Farcomeni A , Milanese A , et al. Statins and major adverse limb events in patients with peripheral artery disease: a systematic review and meta‐analysis. Thromb Haemost. 2020;120:866‐875. 32369857 10.1055/s-0040-1709711

[54] Kokkinidis DG , Arfaras‐Melainis A , Giannopoulos S , et al. Statin therapy for reduction of cardiovascular and limb‐related events in critical limb ischemia: a systematic review and meta‐analysis. Vasc Med. 2020;25:106‐117. 31964311 10.1177/1358863X19894055

[55] Yu W , Wang B , Zhan B , et al. Statin therapy improved long‐term prognosis in patients with major non‐cardiac vascular surgeries: a systematic review and meta‐analysis. Vascul Pharmacol. 2018;109:1‐16. 29953967 10.1016/j.vph.2018.06.015

[56] Parmar GM , Novak Z , Spangler E , et al. Statin use improves limb salvage after intervention for peripheral arterial disease. J Vasc Surg. 2019;70:539‐546. 30718113 10.1016/j.jvs.2018.07.089

[57] Sato Y , Uzui H , Aiki Y , et al. Effects of PCSK9 inhibitor on adverse limb outcomes in patients with critical limb ischemia. Eur Heart J. 2020;41:ehaa946.2375.

[58] Kaneko M , Fujihara K , Harada MY , et al. Rates and risk factors for amputation in people with diabetes in Japan: a historical cohort study using a nationwide claims database. J Foot Ankle Res. 2021;14:29. 33836779 10.1186/s13047-021-00474-8 PMC8034178

[59] Golledge J , Drovandi A , Rowbotham S , Velu R , Quigley F , Jenkins J . Control of modifiable risk factors and major adverse cardiovascular events in people with peripheral artery disease and diabetes. World J Diabetes. 2021;12:883‐892. 34168735 10.4239/wjd.v12.i6.883 PMC8192253

[60] Kołpa M , Grochowska A , Kubik B , Stradomska K . Lifestyle, metabolic compensation in patients with type 2 diabetes mellitus and the risk of chronic disease complications. Clin Diabetol. 2018;7:151‐158.

[61] Fan RR , Gibson AK , Smeds MR , Zakhary E . Impact of socioeconomic status on major amputation in patients with peripheral vascular disease and diabetes mellitus. Ann Vasc Surg. 2022;79:388. 10.1016/j.avsg.2022.03.035 35398196

[62] Caruso P , Scappaticcio L , Maiorino MI , Esposito K , Giugliano D . Up and down waves of glycemic control and lower‐extremity amputation in diabetes. Cardiovasc Diabetol. 2021;20:135. 34229673 10.1186/s12933-021-01325-3 PMC8261935

[63] Boyko EJ , Zelnick LR , Braffett BH , et al. Risk of foot ulcer and lower‐extremity amputation among participants in the diabetes control and complications trial/epidemiology of diabetes interventions and complications study. Diabetes Care. 2022;45:357‐364. 35007329 10.2337/dc21-1816 PMC8914413

[64] Rodionov RN , Peters F , Marschall U , L'Hoest H , Jarzebska N , Behrendt CA . Initiation of SGLT2 inhibitors and the risk of lower extremity minor and major amputation in patients with type 2 diabetes and peripheral arterial disease: a health claims data analysis. Eur J Vasc Endovasc Surg. 2021;62:981‐990. 34782230 10.1016/j.ejvs.2021.09.031

[65] Khouri C , Cracowski JL , Roustit M . SGLT‐2 inhibitors and the risk of lower‐limb amputation: is this a class effect? Diabetes Obes Metab. 2018;20:1531‐1534. 29430814 10.1111/dom.13255

[66] Canavan RJ , Unwin NC , Kelly WF , Connolly VM . Diabetes‐ and nondiabetes‐related lower extremity amputation incidence before and after the introduction of better organized diabetes foot care: continuous longitudinal monitoring using a standard method. Diabetes Care. 2008;31:459‐463. 18071005 10.2337/dc07-1159

[67] Rammos C , Steinmetz M , Lortz J , et al. Peripheral artery disease in Germany (2009–2018): prevalence, frequency of specialized ambulatory care and use of guideline‐recommended therapy ‐ a population‐based study. Lancet Reg Health Eur. 2021;5:100113. 34557822 10.1016/j.lanepe.2021.100113 PMC8454876

[68] Shu J , Santulli G . Update on peripheral artery disease: epidemiology and evidence‐based facts. Atherosclerosis. 2018;275:379‐381. 29843915 10.1016/j.atherosclerosis.2018.05.033 PMC6113064

[69] Balletshofer B , Ito W , Lawall H , et al. Position paper on the diagnosis and treatment of peripheral arterial disease (PAD) in people with diabetes mellitus. Exp Clin Endocrinol Diabetes. 2019;127:S105‐S113. 31860931 10.1055/a-1018-9250

[70] Bonaca MP , Hamburg NM , Creager MA . Contemporary medical Management of Peripheral Artery Disease. Circ Res. 2021;128:1868‐1884. 34110910 10.1161/CIRCRESAHA.121.318258

[71] Narcisse DI , Katzenberger DR , Gutierrez JA . Contemporary medical therapies for patients with peripheral artery disease and concomitant type 2 diabetes mellitus: a review of current evidence. Curr Cardiol Rep. 2022;24:567‐576. 35201560 10.1007/s11886-022-01677-6

[72] Bhagirath VC , Nash D , Wan D , Anand SS . Building your peripheral artery disease toolkit: medical Management of Peripheral Artery Disease in 2022. Can J Cardiol. 2022;38:634‐644. 35151781 10.1016/j.cjca.2022.02.004

[73] Noskin GA , Rubin RJ , Schentag JJ , et al. The burden of Staphylococcus aureus infections on hospitals in the United States: an analysis of the 2000 and 2001 Nationwide inpatient sample database. Arch Intern Med. 2005;165:1756‐1761. 16087824 10.1001/archinte.165.15.1756

